# Multi‐ancestral origin of intestinal tumors: Impact on growth, progression, and drug efficacy

**DOI:** 10.1002/cnr2.1459

**Published:** 2021-07-10

**Authors:** Alyssa A. Leystra, Brock J. Gilsdorf, Amanda M. Wisinger, Elise R. Warda, Shanna Wiegand, Christopher D. Zahm, Kristina A. Matkowskyj, Dustin A. Deming, Naghma Khan, Quincy Rosemarie, Chelsie K. Sievers, Alexander R. Schwartz, Dawn M. Albrecht, Linda Clipson, Hasan Mukhtar, Michael A. Newton, Richard B. Halberg

**Affiliations:** ^1^ McArdle Laboratory for Cancer Research, Department of Oncology University of Wisconsin‐Madison School of Medicine and Public Health Madison Wisconsin USA; ^2^ Division of Gastroenterology and Hepatology University of Wisconsin‐Madison School of Medicine and Public Health Madison Wisconsin USA; ^3^ Department of Pathology and Laboratory Medicine University of Wisconsin‐Madison School of Medicine and Public Health Madison Wisconsin USA; ^4^ University of Wisconsin Carbone Cancer Center University of Wisconsin‐Madison School of Medicine and Public Health Madison Wisconsin USA; ^5^ Division of Hematology and Oncology, Department of Medicine University of Wisconsin‐Madison School of Medicine and Public Health Madison Wisconsin USA; ^6^ Department of Dermatology University of Wisconsin Madison Wisconsin USA; ^7^ Department of Statistics University of Wisconsin‐Madison Madison Wisconsin USA; ^8^ Department of Biostatistics and Medical Informatics University of Wisconsin‐Madison School of Medicine and Public Health Madison Wisconsin USA

**Keywords:** colorectal cancer, drug efficacy, heterotypic tumors with a multi‐ancestral origin/architecture, invasiveness, tumor origin

## Abstract

**Background:**

Data are steadily accruing that demonstrate that intestinal tumors are frequently derived from multiple founding cells, resulting in tumors comprised of distinct ancestral clones that might cooperate or alternatively compete, thereby potentially impacting different phases of the disease process.

**Aim:**

We sought to determine whether tumors with a multi‐ancestral architecture involving at least two distinct clones show increased tumor number, growth, progression, or resistance to drug intervention.

**Methods:**

Mice carrying the *Min* allele of *Apc* were generated that were mosaic with only a subset of cells in the intestinal epithelium expressing an activated form of PI3K, a key regulatory kinase affecting several important cellular processes. These cells were identifiable as they fluoresced green, whereas all other cells fluoresced red.

**Results:**

Cell lineage tracing revealed that many intestinal tumors from our mouse model were derived from at least two founding cells, those expressing the activated PI3K (green) and those which did not (red). Heterotypic tumors with a multi‐ancestral architecture as evidenced by a mixture of green and red cells exhibited increased tumor growth and invasiveness. Clonal architecture also had an impact on tumor response to low‐dose aspirin. Aspirin treatment resulted in a greater reduction of heterotypic tumors derived from multiple founding cells as compared to tumors derived from a single founding cell.

**Conclusion:**

These data indicate that genetically distinct tumor‐founding cells can contribute to early intratumoral heterogeneity. The coevolution of the founding cells and their progeny enhances colon tumor progression and impacts the response to aspirin. These findings are important to a more complete understanding of tumorigenesis with consequences for several distinct models of tumor evolution. They also have practical implications to the clinic. Mouse models with heterogenous tumors are likely better for predicting drug efficacy as compared to models in which the tumors are highly homogeneous. Moreover, understanding how interactions among different populations in a single heterotypic tumor with a multi‐ancestral architecture impact response to a single agent and combination therapies are necessary to fully develop personalized medicine.

## 1 INTRODUCTION

Many cancers are genetically quite heterogeneous. This intratumoral diversity is a serious clinical concern, as it contributes to tumor progression, chemotherapy resistance, tumor recurrence, and poor clinical prognosis.^1^, ^2^, ^3^, ^4^, ^5^ Heterogeneous tumors readily adapt to a variety of insults, including immune surveillance, nutrient deprivation, and chemotherapy.^5^, ^6^ Within a single tumor, genetically diverse clones can interact to the advantage of one or more clones. For example, the cooperation of co‐evolved clones can result in increased tumor growth.^7^, ^8^ In addition, minor tumor subclones can promote the invasion and metastasis of other subclones.^9^, ^10^ Diverse populations within a tumor might even mediate chemotherapy resistance in a non‐cell autonomous manner.^11^ Therefore, understanding tumor evolution and various sources of genetic heterogeneity will lead to a better understanding of tumor evolution, and might lead to the development of better animal models to determine the efficacy of new approaches for cancer prevention and therapy.

**FIGURE 1:**
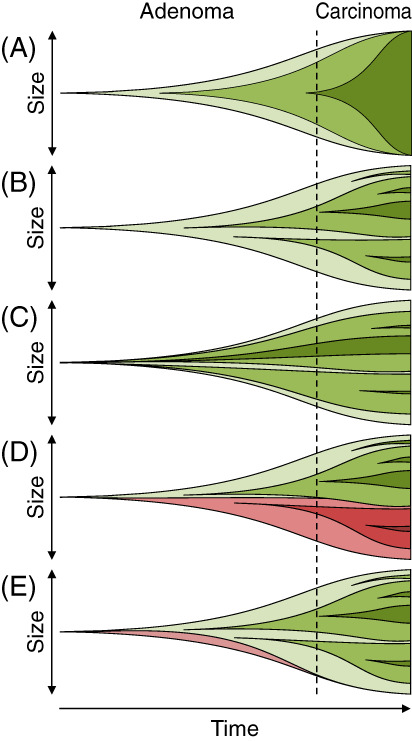
Intratumoral heterogeneity can arise via multiple mechanisms. Various models of tumor evolution have been proposed over the years. In (A) clonal sweep, (B) cancer punctuated equilibrium, and (C) Big Bang postulate that each tumor is derived from a single founding cell. Distinct clones are represented by different shades of green and red. In the clonal sweep model, a single founder acquires a mutation that transforms it from a normal to neoplastic state. As the tumor grows, different progeny acquire additional mutations. A mutation can give a progeny cell and its daughters such a selective advantage over other progeny cells such that they soon “sweep” through a tumor to become a predominant clone, that is, population. This cycle continues in a stepwise fashion until a sufficient number of mutations accumulate such that a benign tumor becomes a cancer. A cancer that forms through this process would be highly homogenous as represented in this schematic with a single cell population shaded dark green (far right). In the cancer punctuated equilibrium model and Big Bang model, waves of mutations occur in progeny cells nearly simultaneously, but the different clones co‐evolve together. The difference in the two models is the timing with sequential waves occurring over time in the cancer punctuated equilibrium model and a single wave occurring very early in tumorigenesis in the Big Bang model. The tumors from these models would be highly heterogeneous as represented in this schematic with multiple cell populations shaded different colors of green (far right). In contrast, (D) the multi‐ancestral model postulates that some tumors are derived from multiple founding cells (red and green). Additional mutations can occur in these founders and their progeny cells as they evolve. Tumors from this model are again highly heterogenous with diversity a reflection of origin from multiple founding cells and evolution as represented in this schematic with multiple cell populations represented by different shades of red and green (far right). (E) A tumor derived from multiple founding cells might not remain heterogeneous. A mutant clone with an alteration in key oncogenic driver like PI3K (green) could drive a sweep eliminating one founding cell and its progeny cells. This possibility was specifically tested in this study

A number of models for tumor evolution have been postulated over the years. A longstanding model is the clonal sweep model. It advanced the notion that a tumor emerged following the neoplastic transformation of a single tumor‐founding cell and that the tumor progressed from a benign to malignant state as progeny acquired new molecular alterations. Defects in DNA repair, telomere maintenance, and chromosome segregation can result in the slow accumulation of point mutations, larger‐scale genomic rearrangements, and copy number alterations. About 60% of colorectal cancers develop through a chromosomal instability pathway and 15% exhibit a high frequency of microsatellite instability.^12^, ^13^ The newly acquired mutation was postulated to provide the clone with a strong selective advantage and thereby force a “sweep” such that the clone with additional alterations is predominant in the tumor (Figure 1(A)).^14^, ^15^ Such a tumor would be highly homogeneous, so this model fails to account for the high degree of intratumoral heterogeneity that is frequently observed.

Other models do account for intratumoral heterogeneity, including the cancer punctuated equilibrium model, the Big Bang model, and the multi‐ancestral model. The cancer punctuated equilibrium model postulates that each cancer is initiated from a single founder but the progeny rapidly acquire molecular changes and the newly formed clones gradually equilibrate over long periods of time until the next burst of molecular changes (Figure 1(B)).^16^, ^17^ Alternatively, emerging data indicate that some tumors might be “born bad” instead of “born good and become bad.” The Big Bang model postulates a burst of molecular changes occurs quite early and that distinct clones co‐evolve over time (Figure 1(C)).^18^ However, an early burst of mutations is unnecessary to explain the existence of several prominent clones in a single tumor if a tumor can form from multiple founder cells instead of a single founder as reviewed previously (Figure 1(D)).^19^ Numerous studies have demonstrated that some tumors from mice and humans have a multi‐ancestral architecture.^20^, ^21^, ^22^, ^23^, ^24^, ^25^, ^26^, ^27^, ^28^, ^29^, ^30^, ^31^, ^32^, ^33^, ^34^

While multi‐ancestral adenomas and adenocarcinomas have been identified in both sporadic and familial cases of colorectal cancer in humans, previous studies did not address the fate of ancestral clones that carry mutations in key tumor driver genes such as *KRAS*, *PIK3CA* encoding PI3K, or *TP53*, which are commonly mutated in colorectal cancers.^15^, ^35^, ^36^ Expression of a tumor driver gene within a single ancestral clone might result in a clonal sweep, eliminating other less fit clones that contribute to a tumor. Therefore, while the tumors in animal models studied previously were frequently derived from multiple tumor‐founding cells, these results may have little clinical impact considering that most human colon cancers acquire at least one of these driver mutations in a subset of tumor cells as a tumor progresses from a benign to the malignant state.^37^

Recently, activated PI3K signaling has been shown to drive rapid tumor initiation, growth, and progression within the mammalian colon of transgenic mice, indicating that PI3K is a key tumor driver within the colon.^38^, ^39^, ^40^ The transgenic mice expressed a constitutively active form of PI3K in which the regulatory domain was fused to the catalytic domain. PI3K transmits signals from various transmembrane growth factor receptors through a kinase cascade to nuclear transcription factors as reviewed previously.^41^ PI3K initiates this signaling pathway through the phosphorylation of phosphatidylinositol 4,5‐bisphosphate (PIP2) to phosphatidylinositol 3,4,5‐trisphosphate (PIP3). PIP3 then activates the serine/threonine kinase AKT, which phosphorylates multiple downstream targets responsible for a wide variety of vital cellular functions. One of the prominent targets is mTOR, a serine/threonine kinase that is an important regulator of cell growth and metabolism. This kinase then mediates activation of the eukaryotic translation initiation factor 4E‐binding protein (4E‐BP1) and the p70S6 ribosomal kinase (S6) that are involved in protein synthesis.

We use a similar transgenic mouse model in this study. The distal small intestine and colon are a mosaics composed of cells expressing the activated form of PI3K and those that do not. The two populations are distinguishable because the former fluoresce green, whereas the latter fluoresce red. This model allowed us to determine whether activation of a strong tumor driver like PI3K within a subset of tumor‐founding cells was sufficient to drive an early clonal sweep or whether tumors would maintain early diversity through tumor growth and progression. The results underscore the importance of early genetic diversity arising from multiple tumor‐founding cells as it impacts many facets of tumor biology including initiation, growth, progression, and response to intervention.

## 2 METHODS

### 2.1 Mice

Animal studies were conducted under protocols approved by the School of Medicine and Public Health Institutional Animal Care and Use Committee at the University of Wisconsin in compliance with policies established by the Office of Laboratory Animal Welfare at the National Institutes of Health. All animals were housed in a specific pathogen‐free facility and fed a standard chow diet containing 4% fat. Genotyping was performed by PCR as described by the suppliers of the animals. FVB.Fabp1‐Cre^+^,^42^ C57BL6/J (B6).mT/mG^+^,^43^ B6.*Apc* ^*Min*/+^,^44^ and B6.Pi3k*^45^ animals were maintained as previously described.^32^ FVB.Fabp1‐Cre^+^ mice were backcrossed to B6 mice for 10 generations to generate B6.Fabp1‐Cre^+^ congenic mice. Mice were interbred to create experimental animals and controls. Additional information on the breeding scheme is available in the Supplementary Methods. Fabp1‐Cre^+^ mT/mG^+^ Pik3ca*^+^ *Apc* ^*Min*/+^ mice were considered experimental mice to assess clonal architecture and its impact on tumor response to aspirin or GDC‐0941, whereas Fabp1‐Cre^+^ mT/mG^+^ *Apc* ^*Min*/+^, mT/mG^+^ Pik3ca*^+^ *Apc* ^*Min*/+^, and mT/mG^+^ *Apc* ^*Min*/+^ mice were *Apc* ^*Min*/+^. Comparisons were made to ensure the observed effects were not due to Fabp1‐Cre^+^ or the mT/mG^+^ transgene (Supplemental Tables S1–S5).

### 2.2 Aspirin treatment

Stock females were bred to stock males and checked daily for a visible plug. Once a plug was detected, the females were removed from the males and placed on either the Teklad Global 2019 diet (Envigo) as a control or else the Teklad Custom Aspirin diet (Envigo; TD.180479; 32 ppm) with their progeny continuing to receive the diet fed to the females until the study was terminated. Between 30 and 40 days of age, the aspirin‐treated and non‐treated control mice underwent colonoscopy as described previously.^46^ The mice were scoped once a week with most being scoped at least four times. Mice were dissected when moribund or at 60 days of age.

### 2.3 GDC‐0941 treatment

Between 40 and 45 days of age, the experimental mice were treated with GDC‐0941 (Selleckchem; 100 μg/g body weight; intraperitoneal injection; 1 injection each day for 14 days). After treatment, the experimental mice (2 mice; 1 male and 1 female) that were treated with GDC‐0941 and control mice (6 mice; 2 females and 4 males) that were treated with vehicle alone were euthanatized, dissected, and imaged. The number of tumors was scored and each tumor was analyzed to determine its clonal origin/architecture.

### 2.4 Whole tissue imaging

Intestinal tracts were removed at necropsy, opened longitudinally, rinsed with PBS, fixed in 4% paraformaldehyde for 2 days at 4°C, and then stored in 70% ethanol at 4°C. To document the pattern of mosaicism, the small intestine and colon were mounted in 0.4% agarose and imaged with a Nikon Eclipse TI‐S microscope or Zeiss AxioPlan II.^32^

### 2.5 Whole tissue patchwork analysis

The intestinal epithelium in experimental and control mice is a mosaic of GFP‐positive and of RFP‐positive crypts. The ability to detect multi‐ancestral tumors depends on the pattern of mosaicism. To quantify the mosaicism, whole‐tissue fluorescent images that were taken on a Nikon Eclipse TI‐S microscope were exported from ND2 files to single channel TIFF files prior to analysis. All images were processed using a macro for the Fiji image‐processing package of ImageJ to ensure consistency. Additional details on the image‐processing steps are available in the Supplemental Methods. For quality control, binary images were compared to color overlays; when necessary, the images were manually manipulated to more accurately reflect the patchwork of the tissue.

A variegation score was assigned to each binary image (Supplemental Figure S1). The length of the perimeter of the patches of green pixels was assumed to be proportional to the number of crypts that were adjacent to at least one crypt of a different color. However, some of the perimeter would be positioned on the edge of the tissue instead of adjacent to crypts of a different color. To correct for this, it was assumed that the proportion of the total perimeter of the tissue that was composed of green pixels would be approximately equal to the proportion of the total area of the tissue that was composed of green pixels. Therefore, the perimeter of green pixels was corrected by subtracting out the appropriate proportion of the total tissue perimeter. The corrected perimeter was then normalized to the square root of the total area of the tissue to determine the final variegation score.

$$\text{Variegation score}=\frac{\text{perimeter}\left(\text{green}\right)-\left[\text{proportion}\left(\text{green}\right)*\text{perimeter}\left(\text{tissue}\right)\right]}{\sqrt{\text{area}\left(\text{tissue}\right)}}$$

A low variegation score indicates that there is limited mosaicism, for example, the proximal region of the small intestine where all cells fail to express Cre recombinase from the transgene, whereas a high variegation score indicates that there is comprehensive mosaicism, for example, the colon where half of the cells fail to express Cre recombinase (RFP^+^) and half the cells express Cre recombinase (GFP^+^).

### 2.6 Immunohistochemistry and antibodies

To determine the clonal origin/architecture, each tumor was carefully analyzed histologically. Tumors that were easily visible without the use of a dissecting microscope (usually >2 mm in maximum diameter) were excised, embedded in paraffin, and cut into 5 μm sections. Every 20th section was stained with hematoxylin and eosin (H&E).

To determine whether cells from distinct lineages were neoplastic, slides of interest were stained by immunofluorescence for β‐catenin as described previously^39^ as well as tdTomato and EGFP using the following conditions: permeabilization in 0.5% Tween20 solution in PBS; antigen presentation in 0.5% NaBH_4_ solution in PBS for 10 min; blocking in 5% dehydrated milk in PBS for at least 1 h; incubation in anti‐tdTomato (1:200 anti‐RFP Rabbit pAB Rockland; RL600‐401‐379, VWR Scientific, Radnor PA) and anti‐EGFP (1:1000 Living Colors anti‐EGFP Mouse mAB; 632 569, Clontech Laboratories, Fitchburg WI) primary antibodies overnight; incubation in anti‐rabbit (1:1000 Alexa Fluor 568 anti‐rabbit IgG [H + L] Goat mAB; A11011, Invitrogen, Carlsbad, CA) and anti‐mouse (1:1000 Alexa Fluor 488 anti‐mouse IgG2a Goat mAB; A21131, Invitrogen) secondary antibodies overnight; and staining of nuclei with 4′,6‐diamidino‐2‐phenylindole (DAPI) by applying ProLong Gold with DAPI mounting media (P36931, Invitrogen).

Images of H&E and immunohistochemical stained slides were acquired on a Nikon Eclipse TI‐S microscope using a halogen light source, no color filters, and either a 4× or 10× objective lens. Images of immunofluorescent stained slides were acquired on either a Zeiss AxioPlan II or Nikon Eclipse TI‐S microscope as previously described with either a 10× or 20× objective lens.^32^

The phenotype of each tumor was determined by a board‐certified pathologist (KAM). H&E‐stained slides were examined to identify neoplastic cells and areas of invasion and then compared to the immunofluorescent stained slides to determine which neoplastic cells were red and which neoplastic cells were green. Homotypic tumors were composed entirely of either RFP+ neoplastic cells or GFP+ neoplastic cells, whereas heterotypic tumors with a multi‐ancestral architecture were composed of a mixture of RFP‐positive and GFP‐positive neoplastic cells.

### 2.7 Organoid generation, imaging, and scoring

Tumor samples were isolated for organoid culture based on an adaptation of a published protocol.^47^ Mice were euthanized, the intestinal tract was removed, and tumors were isolated. Tumors were rinsed in ice‐cold PBS, chopped into small pieces, suspended in chelation buffer (2 mM EDTA, 5.6 mM Na_2_HPO_4_, 8 mM KH_2_PO_4_, 96.2 mM NaCl, 1.6 mM KCl, 43.4 mM sucrose, 54.9 mM D‐sorbital, and 0.5 mM DL‐Dithiothreitol) in a 15 m tube for 1 h on ice. After the incubation, samples were rinsed with chelation buffer and PBS. The samples were then suspended in DMEM containing 2.5% FBS, 50 U/ml Penicillin, 50 μg/ml Streptomycin, 1 mg/ml Collagenase Type XI (Sigma C9697), and 1.25 mg/ml Dispase II (Sigma D4963), placed for 45 min at 37°C, and shaken vigorously every 15 min. The supernatant from the digest was transferred to a clean 15 ml tube and cells were pelleted. The cells were washed three times and resuspended in cold PBS at a concentration of 600 cells/μl.

Cell suspensions were mixed 1:1 with Growth‐Factor Reduced Matrigel (Corning #354230) and 50 μl drops were placed in the center of wells in a 24 well plate. All samples were plated in duplicate. Matrigel was allowed to solidify for 15 min at 37°C. Wells were cultured with 500 μl of advanced DMEM containing 50 U/ml Penicillin, 50 μg/ml Streptomycin, 1× Glutamax (Gibco), 1× HEPES, 1× B‐27 Supplement (Gibco), 1× N‐2 Supplement (Gibco), and 500 ng/ml mouse recombinant EGF. Media was changed every other day.

To collect longitudinal data on growth patterns of tumor cells, six tumors were isolated as described above and plated in duplicate: two in which all neoplastic epithelial cells expressed tdTomato (“homotypic red”), two in which all neoplastic epithelial cells expressed EGFP (“homotypic green”), and two composed of a mixture of epithelial cells expressing tdTomato and EGFP (“heterotypic”). In addition, cells from each homotypic red tumor were mixed 50:50 with cells from each homotypic green tumor and plated in duplicate.

A total of 299 organoids was imaged daily for 7 days. To collect longitudinal data, three random areas were selected from each well and imaged on day 0 before organoid formation; these areas were then imaged daily on days 1–7. White light, red fluorescent, and green fluorescent images were collected with a Nikon Eclipse TI‐S microscope with a 4× objective lens as previously described.^32^

To determine growth patterns, images were used to measure the maximum diameter of every organoid that was observed throughout the longitudinal experiment at each time point. The diameter growth rate for each organoid was calculated as the change in maximum diameter from the first time point to the final time point the organoid was observed, divided by the number of days the organoid was observed.

### 2.8 Tumor engrafting

Tumor cells were isolated as described above. After final washes, tumor samples were engrafted onto the flank of naïve B6 recipients. Minced tumors, cells from the digest, cells from sorting, or organoids were mixed at a 1:1 ratio with Matrigel (Corning #356234). For sorting, cells were suspended in PBS + 2% FBS + 1 mM EDTA, sorted into ADF + 2% FBS, spun down, and then resuspended in culture medium prior to injection. The samples consisting of 3 × 10^5^ to 3 × 10^6^ cells in a volume of less than 400 μl were injected subcutaneously in the flank. The recipients were monitored weekly to assess tumor development and growth. The tumors were measured using veneer calipers. When engrafted tumors reached a volume of 2 cm^3^, the recipients were euthanized.

### 2.9 Statistical analysis

Differences in tumor multiplicities and sizes between groups of mice were tested for statistical significance using the two‐sided Wilcoxon rank sum test. Proportions of tumors and clones within tumors that were invasive were tested with a two‐sided Fisher's exact test.

## 3 RESULTS

### 3.1 Transgenic animals facilitate labeling cells prior to tumor initiation

To trace individual clones through tumor initiation, growth, and progression, we utilized transgenic animals that stably express reporter genes to differentially label clones in the intestinal tract. In Fabp1‐Cre^+^ mT/mG^+^ double transgenic mice, Cre recombinase is expressed in approximately 50% of the epithelial cells in the distal small intestine and colon during embryogenesis; cells are then labeled by a reporter that expresses either a derivative of a red fluorescent protein (tdTomato) in the absence of Cre‐mediated recombination or an enhanced green fluorescent protein (EGFP) following Cre‐mediated recombination. In this way, clones are labeled prior to tumor initiation. The validity of this model for tracing ancestral clones in tumors was previously demonstrated in *Apc* ^*Min*/+^ mice, which develop tumors throughout the entire length of the intestinal tract.^32^ Importantly, the labeled crypts in the normal intestinal epithelium of Fabp1‐Cre^+^ mT/mG^+^ *Apc* ^*Min*/+^ mice are either wholly red or wholly green, the pattern of mosaicism that is established during embryogenesis is stable over time, and some (51%; 32/63) tumors were multi‐ancestral as they were composed of a mixture of wholly red or wholly green dysplastic crypts.^32^

**FIGURE 2:**
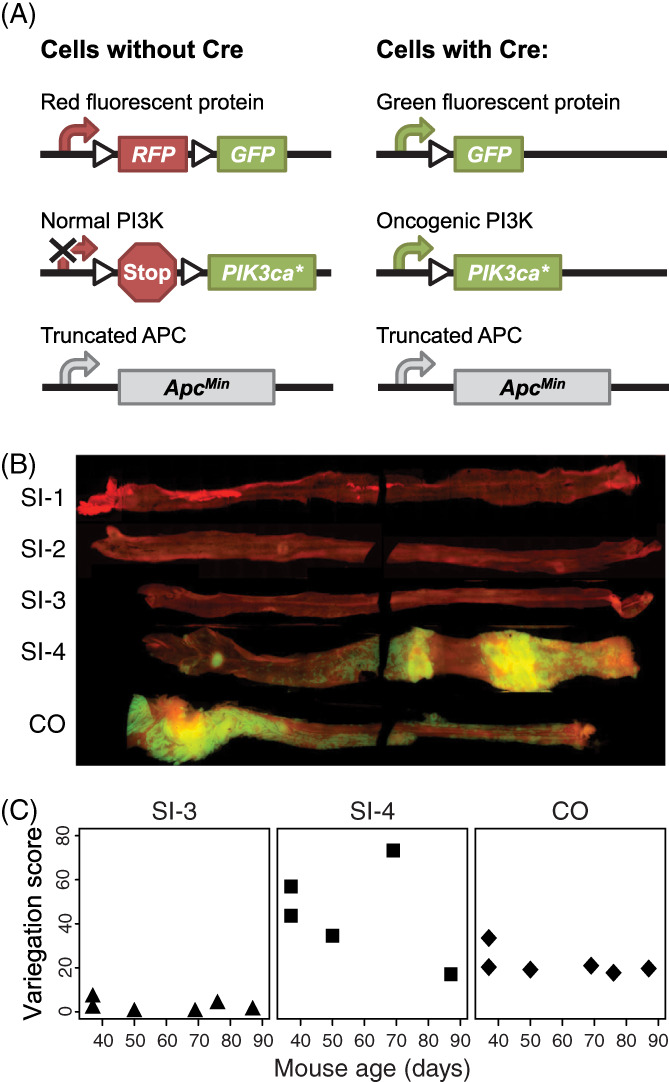
The distal small intestine and colon of Fabp1‐Cre^+^ mT/mG^+^ Pik3ca*^+^ *Apc* ^*Min*/+^ mice were a variegated pattern of fluorescently labeled cells. (A) The Fabp1‐Cre^+^ mT/mG^+^ Pik3ca*^+^ *Apc* ^*Min/+*^ mice carry three transgenes: one transgene (Fabp1‐Cre) expresses Cre recombinase from the fatty acid binding protein promoter, the second transgene (mT/mG) is a reporter Cre recombinase activity, the third transgene (Pik3ca*) encodes a constitutively activated form of PI3K which a kinase that controls several different cellular processes. Cre recombinase was expressed in about half of the epithelial cells of the distal small intestine and colon of Fabp1‐Cre^+^ mT/mG^+^ Pik3ca*^+^ *Apc* ^*Min/+*^ mice. Cells without Cre recombinase expressed tdTomato (RFP). In cells expressing Cre recombinase, however, recombination at *loxP* sites (triangles) within two transgenes led to expression of GFP in place of tdTomato and expression of a constitutively activated PI3K oncoprotein. All cells in the mice lacked one functional allele of *Apc*. (B) The intestinal tract was removed from an experimental mouse. The small intestine was divided into four equal segment (SI‐1 through SI‐4 with SI‐1 being closest to the stomach). The small intestinal segments and colon were opened and imaged with a fluorescence microscope. The distal small intestine (SI‐4) and colon (CO) were therefore a mosaic of tdTomato‐expressing (red) cells with low relative risk of malignant transformation and GFP‐expressing (green) cells with high relative risk of malignant transformation owing to the presence of constitutively activated PI3K oncoprotein. (C) The degree of mosaicism in different regions was assessed by calculating the variegation score. A high score means more mosaicism—smaller intermingled patches of red and green cells. Variegation scores for 3 female and 3 male mice for SI‐4 (where one of the males could not be scored) and colon were each significantly higher than for SI‐3 (*p* = 0.004 and 0.002, respectively, two‐sided Wilcoxon rank sum tests)

To introduce a strong tumor driver within a subset of tumor clones, the Pik3ca* transgene was used. This transgene results in the expression of constitutively activated PI3K within cells that express Cre recombinase.^38^ When combined with the animal model described above, genetically distinct cell lineages are labeled prior to tumor formation (Figure 2(A)). Normal intestinal tissues from Fabp1‐Cre^+^ mT/mG^+^ Pik3ca*^+^ *Apc* ^*Min*/+^ animals were a mixture of tdTomato‐labeled (red) cells harboring a mutant *Apc* allele and EGFP‐labeled (green) cells harboring a mutant *Apc* allele and expressing activated PI3K. As tumors with a multi‐ancestral origin/architecture can only be detected when they arise from crypts expressing different colored labels, and because they arise as a consequence of short‐range interactions among neighboring crypts,^26^ the ability to detect tumors with a multi‐ancestral origin/architecture depends on the degree to which red and green crypts are intermingled, that is, the “variegation” of the tissue. More variegation increases the probability that tumors with a multi‐ancestral origin/architecture will be observed because the tumor founding cells are more likely to be different colors. Therefore, the variegation of each region of intestinal tissue was scored in Fabp1‐Cre^+^ mT/mG^+^ Pik3ca*^+^ *Apc* ^*Min*/+^ mice. A high score indicates more intermingling (Figure S1). Variegation was essentially non‐existent in the proximal small intestine (SI‐1 and SI‐2) or very low (SI‐3) because the Fabp1 promoter driving Cre recombinase fails to be expressed or it is expressed in few cells in these regions. By contrast, variegation significantly increased in the distal small intestine (SI‐4) and colon (CO) (Figure 2(B,C)) because the Fabp1 promoter is expressed in many cells in these regions. The variegation score was less than 7 in SI‐3, whereas it ranged from 17‐73 in SI‐4 and 18‐34 in CO (Figure 2(C)).

### 3.2 Constitutive activation of PI3K increases tumor number and size

**FIGURE 3:**
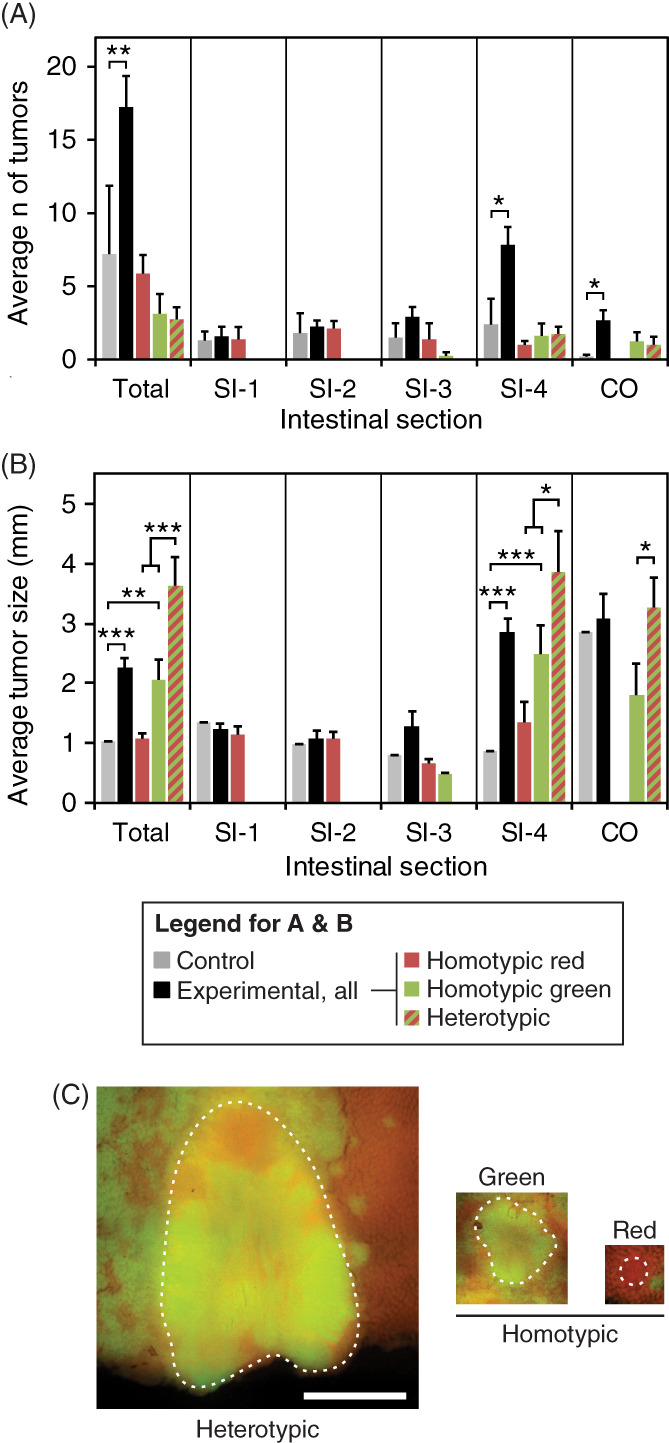
Constitutive activation of PI3K resulted in a tumor‐autonomous increase in number and size. (A) The average number of tumors per mouse was higher in intestinal regions (SI‐4 and colon) of Fabp1‐Cre^+^ mT/mG^+^ Pik3ca*^+^ *Apc* ^*Min/+*^ mice where PI3K was constitutively activated than in *Apc* ^*Min/+*^ controls (two‐sided Wilcoxon rank sum tests: total *p* = 0.001; SI‐1 *p* = 0.6; SI‐2 *p* = 0.1; SI‐3 *p* = 0.2; SI‐4 *p* = 0.004; CO *p* = 0.004). The number of tumors from Fabp1‐Cre^+^ mT/mG^+^ Pik3ca*^+^ *Apc* ^*Min*/+^ mice which were entirely composed of tdTomato‐expressing cells (red) was similar to the total number of tumors from *Apc* ^*Min*/+^ controls (gray; two‐sided Wilcoxon rank sum tests: total *p* = 0.2; SI‐1 *p* = 1.0; SI‐2 *p* = 0.06; SI‐3 *p* = 0.8; SI‐4 *p* = 0.5; CO *p* = 0.2). Thus, the increase in tumor number within these animals can be explained by tumors in which PI3K is constitutively activated (green homotypic or heterotypic). Some tumors within these mice could not be classified. Error bars represent SE of the mean. (B) Tumors were larger in regions (SI‐4 and CO) where PI3K was constitutively activated compared to controls (two‐sided Wilcoxon rank sum tests: total *p* < 0.001; SI‐1 *p* = 0.6; SI‐2 *p* = 0.9; SI‐3 *p* = 0.6; SI‐4 *p* < 0.001; CO *p* = 0.8). Tumor size (maximum diameter) was similar between tumors from Fabp1‐Cre^+^ mT/mG^+^ Pik3ca*^+^ *Apc* ^*Min*/+^ mice that were entirely composed of tdTomato‐expressing cells (red) and those from *Apc* ^*Min/+*^ controls (gray; two‐sided Wilcoxon rank sum tests: total *p* = 0.8; SI‐1 *p* = 0.5; SI‐2 *p* = 0.9; SI‐3 *p* = 0.4; SI‐4 *p* = 0.5). Thus, the increase in tumor size within these animals can be explained by tumors in which PI3K is constitutively activated (green; two‐sided Wilcoxon rank sum tests compared to *Apc* ^*Min/+*^ control tumors: total *p* = 0.004; SI‐3 *p* = 0.1; SI‐4 *p* < 0.001; CO *p* = 0.8). Additionally, heterotypic tumors with a multi‐ancestral architecture were larger than their homotypic counterparts (two‐sided Wilcoxon rank sum tests: total *p* < 0.001; SI‐4 *p* = 0.02; CO *p* = 0.03). Error bars represent SE of the mean. (C) Examples of a heterotypic tumor with a multi‐ancestral origin, a homotypic green tumor, and a homotypic red tumor are shown to scale. Twelve Fabp1‐Cre^+^ mT/mG^+^ Pik3ca*^+^ *Apc* ^*Min*/+^ mice (7 female and 5 male) and 10 *Apc* ^*Min*/+^ controls (5 female and 5 male) were used in this study. Tumors are shown at the same magnification; size bar = 2.0 mm

Constitutive activation of PI3K in Fabp1‐Cre^+^ mT/mG^+^ Pik3ca*^+^ *Apc* ^*Min*/+^ mice resulted in increased tumor number compared to littermate controls (17.3 ± 2.1 vs. 7.2 ± 4.7, respectively). A difference in tumor maximum diameter between experimental and controls was also observed (2.3 ± 0.1 mm vs. 1.0 ± 0.1 mm, respectively). These effects were expected based on the results of a previous study.^39^ These differences were only observed within intestinal regions where PI3K was constitutively activated (Figure 3; Supplemental Tables S1 and S2). Fabp1‐Cre^+^ mT/mG^+^ Pik3ca*^+^ *Apc* ^*Min*/+^ mice with PI3K activated developed 3‐fold more tumors on average in the distal quarter of the small intestine (SI‐4) than *Apc* ^*Min*/+^ controls without PI3K activated (7.8 ± 1.2 vs. 2.4 ± 1.8, respectively). Moreover, Fabp1‐Cre^+^ mT/mG^+^ Pik3ca*^+^ *Apc* ^*Min*/+^ mice developed on average 13‐fold more tumors in the colon (CO) *Apc* ^*Min*/+^ controls (2.7 ± 0.7 vs. 0.2 ± 0.1, respectively). Expression of the mT/mG reporter transgene did not significantly change tumor number (Supplemental Table S5).

To determine whether the Pik3ca*‐dependent increase in tumor size was a tumor‐autonomous or systemic effect, the sizes of tumors within Fabp1‐Cre^+^ mT/mG^+^ Pik3ca*^+^ *Apc* ^*Min*/+^ mice were compared. The clonal architecture of a tumor was predicted from fluorescent images of the surface of whole tumors (Figure 3(C)). All (100%; 28/28) tumors were composed entirely of tdTomato‐expressing cells (homotypic red) in SI‐1 and SI‐2 where constitutive activation of PI3K was not expected; other (61%; 45/74) tumors contained EGFP‐expressing epithelial cells (green) in SI‐4 and CO where constitutive activation of PI3K was expected. The size of homotypic red tumors overall (1.1 ± 0.1 mm) and within each intestinal segment (S1‐1, 1.1 ± 0.1 mm; SI‐2, 1.1 ± 0.1 mm, SI‐3, 0.7 ± 0.1 mm, SI‐4, 1.4 ± 0.3 mm; CO, N/A) was similar to the size of tumors in *Apc* ^*Min*/+^ control mice (total, 1.0 ± 0.1 mm; S1‐1, 1.4 ± 0.2 mm; SI‐2, 1.0 ± 0.1 mm, SI‐3, 0.8 ± 0.1 mm, SI‐4, 0.9 ± 0.1 mm; CO, 2.9 ± 0.2 mm) (Figure 3(B), gray vs. red bars; Supplemental Tables S3 and S4). Meanwhile, tumors that contained green cells (heterotypic or homotypic green) were significantly larger than homotypic red tumors (Figure 3(B), green/red hatched and green bars vs. red bars; *p* = 0.01 for tumors within the distal quarter (SI‐4) of the small intestine; Supplemental Table S4, heterotypic total, 3.6 ± 0.5 mm and homotypic green, total 2.1 ± 0.3 mm vs. homotypic red, total 1.1 ± 0.1 mm). Thus, the increase in tumor size in experimental animals can be accounted for by tumors that contain green cells (Figure 3(B,C)). Together, these data indicate that the PI3K‐dependent increase in tumor size was primarily a tumor‐autonomous effect. This finding illustrates a strength of mosaics in which each mouse is its own internal control—the observed phenotype is specific to cells expressing the gene of interest.

### 3.3 Multi‐ancestral tumors are formed despite expression of the Pik3ca* transgene

**FIGURE 4:**
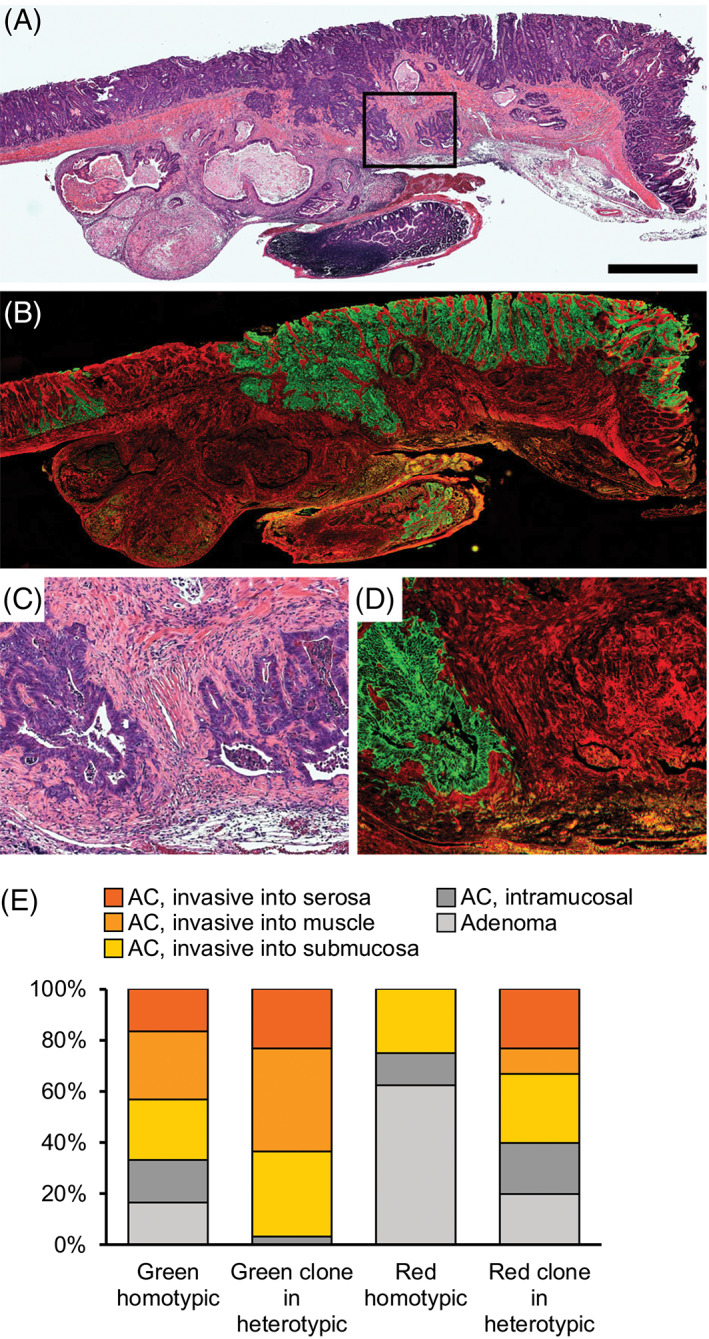
Distinct clones within heterotypic tumors were frequently invasive. (A) Tumors from Fabp1‐Cre^+^ mT/mG^+^ Pik3ca*^+^ *Apc* ^*Min*/+^ mice were excised, embedded in paraffin, and sectioned. Sections were stained hematoxylin and eosin (H&E). Many (75%; 51/68) tumors were adenocarcinomas with clear invasion into and through the muscle layers. A representative is shown. (B) Multiple clones could be identified within heterotypic tumors with a multi‐ancestral architecture by staining for tdTomato (red) and EGFP (green). (C, D) Close examination of neoplastic red and green cells frequently revealed invasion of both clones into the muscle layer. (E) Clones were scored for degrees of invasion. Some clones were not invasive through the lamina propria (shades of gray) and some were (shades of yellow/orange). Both red and green clones within heterotypic tumors were more frequently invasive (green: 29/30 vs. 20/30, *p* = 0.006; red: 18/30 vs. 2/8, *p* = 0.1; Fisher's exact tests) and tended to invade farther than similarly colored clones in homotypic tumors. Size bar for A and B = 1 mm; C and D are 4× enlargements of the area outlined in A

To confirm the presence of multiple neoplastic clones within tumors, 68 large (>2 mm maximum diameter) tumors from Fabp1‐Cre^+^ mT/mG^+^ Pik3ca*^+^ *Apc* ^*Min*/+^ mice were sectioned and stained (Figure 4). H&E slides were reviewed to identify neoplastic cells and areas of invasion, and then tdTomato and EGFP staining was reviewed to determine whether red neoplastic cells, green neoplastic cells, or both were invading. Note that only a limited number of sections of each tumor were examined after histological sectioning, resulting in a potential bias toward falsely classifying heterotypic tumors as homotypic. Overall, 12% (8/68) of sectioned tumors were classified as homotypic red, being composed entirely of tdTomato‐expressing neoplastic cells, and 44% (30/68) were classified as homotypic green, being composed entirely of EGFP‐expressing neoplastic epithelial cells intermingled with tdTomato‐expressing stromal cells. The remaining 44% (30/68) of tumors were heterotypic being composed of a mixture of tdTomato‐ and EGFP‐expressing neoplastic epithelial cells, indicating that multiple clones still contributed to a single tumor even when at least one clone expressed the Pik3ca* transgene prior to tumor initiation (Figure 4(A–D)).

### 3.4 Multi‐ancestral tumor architecture is associated with progression to malignancy

The presence of multiple ancestral clones was strongly associated with the likelihood that a tumor had progressed to an adenocarcinoma; only 6% (1/17) of non‐invasive tumors were heterotypic compared to 57% (29/51) of adenocarcinomas (Table 1; two‐sided Fisher's exact test, *p* < 0.001). We next sought to determine whether the clones were cooperating to their mutual advantage (Figure 4(E)). EGFP‐labeled cells were invasive in 67% (20/30) of homotypic green tumors compared to 97% (29/30) of heterotypic tumors (two‐sided Fisher's exact test, *p* = 0.006). tdTomato‐labeled cells showed a similar trend but the change was not statically significant. They were invasive in 25% (2/8) of homotypic red tumors, which lacked a neoplastic EGFP‐labeled component, and in 60% (18/30) of heterotypic tumors in which the tdTomato‐labeled cells had neoplastic EGFP‐labeled partners (two‐sided Fisher's exact test, *p* = 0.1). In addition, both clones invaded farther in invasive multi‐ancestral tumors than in their homotypic counterparts (Figure 4(E)). Thus, multi‐ancestral architecture is significantly associated with invasion, regardless of whether the clone expressed activated PI3K or not.

**TABLE 1:** Heterotypic tumors with a multi‐ancestral architecture are more invasive than homotypic tumors from Fabp1‐Cre^+^ mT/mG^+^ Pik3ca*^+^ *Apc* ^*Min*/+^ mice

	No. of tumors	
Depth of tumor invasion	Heterogeneous	Homogeneous
Adenoma	0	10
Intramucosal adenocarcinoma	1	6
Invasive adenocarcinoma—Invading into submucosa	12	9
Invasive adenocarcinoma—Invading into muscle	5	8
Invasive adenocarcinoma—Invading to serosa	12	5

### 3.5 Tumors that are composed of some Pik3ca‐mutant cells respond to aspirin and GDC‐0941

**FIGURE 5:**
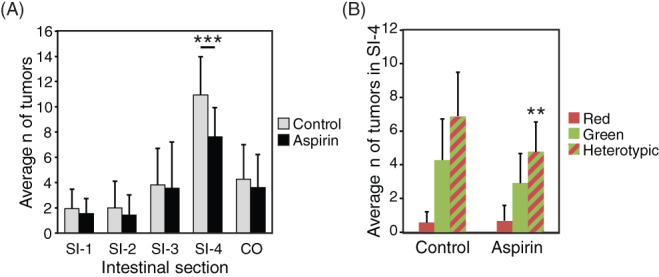
Pik3ca‐mutant tumors respond to low‐dose aspirin. Fabp1‐Cre^+^ mT/mG^+^ Pik3ca*^+^ *Apc* ^*Min*/+^ mice were fed a specialized diet to mimic a low‐dose aspirin regimen in humans and then euthanized to score the number of intestinal tumors in all four regions of the small intestine (SI‐1–SI‐4) and colon (CO). (A) Treated mice developed significantly fewer tumors in SI‐4 than controls (*p* < 0.001). (B) The reduction in this region specifically reflected fewer homotypic green and heterotypic tumors. The change in heterotypic tumors was statistically significant (*p* < 0.05). Data from 23 aspirin‐treated mice (15 female and 8 male) and 18 (10 female and 8 male) controls are shown

Epidemiological studies have found that low‐dose aspirin reduces the risk of colorectal cancer recurrence in patients treated for PIK3CA‐mutant cancers.^48^, ^49^ We tested whether low‐dose aspirin impacted the development of homotypic red, homotypic green, and heterotypic tumors in Fabp1‐Cre^+^ mT/mG^+^ Pik3ca*^+^ *Apc* ^*Min*/+^ mice. Low‐dose aspirin reduced the number of tumors in the most distal quarter of the small intestine in the aspirin‐treated cohort which developed on average 8.1 ± 2.1 as compared to that observed in the non‐treated control cohort which developed on average 11.0 ± 3.0 (Figure 5(A), *p* < 0.001, Wilcoxon rank sum test). The numbers of homotypic green and heterotypic tumors were less in the aspirin‐treated cohort than the non‐treated control cohort (aspirin‐treated, homotypic green, 2.9 ± 1.8 and heterotypic, 4.8 ± 1.8 vs. non‐treated, homotypic green, 4.3 ± 2.4 and heterotypic, 6.9 ± 2.6, respectively), whereas the number of homotypic red was unchanged when comparing the two cohorts (aspirin‐treated, 0.7 ± 0.9 vs. non‐treated, 0.6 ± 0.6) (Figure 5(B)). The effect on heterotypic tumors was statistically significant (*p* = 0.007, Wilcoxon rank sum test). Thus, aspirin appears to inhibit the development of Pik3ca‐mutant tumors in the laboratory mice, recapitulating its effect on PIK3CA‐mutant cancers in humans.

Preliminary data indicate that susceptible cells might be even protected by resistant cells. We treated Fabp1‐Cre^+^ mT/mG^+^ Pik3ca*^+^ *Apc* ^*Min*/+^ mice with GDC‐0941, an inhibitor of PI3K (Supplemental Figure S4) or vehicle. Experimental mice developed a total of 25 tumors: 8 homotypic red, 5 homotypic green, and 12 heterotypic, whereas control mice developed a total of 59 tumors: 6 homotypic red, 30 homotypic green, and 23 heterotypic. Treated mice developed a lower percentage of homotypic green tumors and a higher percentage of red and heterotypic tumors than controls (chi‐square test, *p* = 0.005). The change in the distribution of tumor types was significant when comparing the GDC‐0941 treated cohort to the non‐treated control cohort (chi‐square test, *p* = 0.003).

### 3.6 Organoid generation and tumor grafting permit further analysis of distinct clones

Understanding how the presence of cells from multiple distinct founders impacts tumor biology requires having adequate sample material to more fully characterize molecular features of distinct cell populations. Organoids were easily prepared from 2 homotypic red, 2 homotypic green, and 2 heterotypic tumors from Fabp1‐Cre^+^ mT/mG^+^ Pik3ca*^+^ *Apc* ^*Min*/+^ mice (Supplemental Figure S2). The growth of 299 organoids was followed over a period of 7 days. Interestingly, growth appears to depend on whether the cells were isolated from a homotypic tumor or a heterotypic tumor. The maximum diameter of red organoids started from a red homotypic tumor doubled on average in 18 h, whereas red organoids from a heterotypic tumor doubled on average every 20 h. Similarly, green organoids from homotypic green tumors had a doubling time of 24 h, whereas green organoids from a heterotypic tumor had a doubling time of 21 h. We have successfully grafted homotypic red, homotypic green, and heterotypic tumors from the intestine of Fabp1‐Cre^+^ mT/mG^+^ Pik3ca*^+^ *Apc* ^*Min*/+^ mice onto the flank of naïve B6 recipients. Samples were prepared from intestinal tumors by mincing, generating single cell isolates by digestion, producing organoids, or sorting cells (Supplemental Figure S3(A,B)). Primary tumors and grafts were stained with H&E and by immunohistochemistry for red fluorescence protein, green fluorescence protein, and β‐catenin to compare tumor morphology and composition of the two sample types. Nuclear β‐catenin is a tumor marker in the intestinal tract because the loss of APC activity triggers the stabilization of β‐catenin which then translocates into the nucleus and alters the expression of numerous genes. An example of a comparison is shown (Supplemental Figure 4(B)). The tumor was a homotypic green tumor that was highly invasive. Nuclear β‐catenin was evident in the primary tumor and graft.

## 4 DISCUSSION

This study is the first to investigate multi‐ancestral intestinal cancers with a known oncogenic driver. Despite constitutive activation of PI3K prior to tumor initiation, multiple distinct clones in a single tumor contribute to progression. Tumors with a multi‐ancestral architecture are larger and more invasive than other tumors. Furthermore, co‐evolved clones within heterotypic tumors with a multi‐ancestral architecture are more invasive than within homotypic tumors. Interclonal cooperation can drive tumor maintenance, growth, progression, and response to therapy.^10^ Others have demonstrated that multiple cooperating tumor clones can be essential for tumor propagation^39^ and that non‐dominant clones can promote the survival and metastatic potential of dominant clones.^10^ Thus, multiple co‐evolved ancestral clones appear to cooperate to promote survival, growth, and invasion.

Genetic diversity can arise as a consequence of clonal divergence through molecular alterations. Defects in DNA repair, telomere maintenance, and chromosome segregation can result in the slow accumulation of point mutations, larger‐scale genomic rearrangements, and copy number alterations. About 60% of colorectal cancers develop through a chromosomal instability pathway and 15% exhibit a high frequency of microsatellite instability.^12^, ^13^ Thus, a single transformed cell can give rise to a tumor composed of several distinct clones if molecular alterations do not provide a strong selective advantage and subsequently are unable to drive a clonal sweep. Alternatively, genetic diversity can also arise during tumor initiation if multiple cells become transformed over a short period of time. The resulting tumors are inherently heterogeneous owing to the presence of multiple genetically and phenotypically distinct cells at the earliest point in the development of a tumor. Though many cancers can be derived from multiple founders, intestinal tumors have been studied most thoroughly as described in two reviews.^19^, ^33^ Depending on the biological context and sensitivity of the assay, multi‐ancestral tumors have been observed at frequencies ranging from 10% to 100% of intestinal tumors examined.^22^, ^23^, ^24^, ^25^, ^26^, ^50^, ^51^ However, it was unclear whether multiple founders would co‐evolve as the tumor progressed if any one founder developed a mutation in a strong oncogenic driver (Figure 1(E)). This study clearly demonstrates that the clone carrying an oncogenic driver does not obligate a clonal sweep. In fact, the presence of clones with distinct genotypes appears to facilitate tumor growth and progression.

PI3K activation has often been considered a late event in tumor evolution. Activating mutations in *PIK3CA* are seldom detected in adenomas and are instead restricted to cancers. However, tumors carrying activated PI3K signaling still undergo the traditional adenoma‐to‐adenocarcinoma transition, albeit likely at an increased rate.^52^ In addition, these mutations are often detected within only a subset of tumor cells.^35^ When driver mutations with a significant competitive advantage arise within a subset of tumor cells, some in silico models predict that these cells can rapidly outcompete other nearby clones and thus expand to detectable levels within large tumors.^53^ If PI3K drives clonal sweeps, however, our endogenous tumor model in which PI3K mutations can be present when the tumor initiates should not result in any heterotypic tumors. Contrary to this possibility, tumors were frequently heterotypic. Our data would therefore indicate that the frequent presence of PI3K within a subset of tumor cells might not be a consequence of late arrival but rather an important early event during tumorigenesis.

A surprising observation was that heterotypic tumors were more invasive than homotypic green tumors. The mixture of tumor cells with a risk of malignant transformation (green—fully functional APC activity lost and PI3K activated) and tumor cells with a relatively low risk of malignant transformation (red—fully functional APC activity lost) resulted in a greater invasion of even the green cells. Other studies of clonal architecture made similar observations. Marusyk et al.^10^ demonstrated that tumors can be driven by a sub‐population of cells that does not have higher fitness, but instead stimulates the growth of all tumor cells non‐cell autonomously by inducing tumor‐promoting microenvironmental changes.

Our study has implications for the clinic. The research team of Dr. Liskay reported that a critical mass of Apc‐deficient cells must form to efficiently establish a tumor.^30^ They argued that one way to expand is the coalescence of two independent fields. Our earlier work indicates that it is not coalescence but the recruitment of neighboring cells. Understanding the recruitment process could reveal new targets for prevention. The recruitment process is not disrupted by a founding cell and its progeny cells having a strong driver mutation. Our findings are also likely relevant to treatment. Animal models in which tumors can be heterogeneous are critical to preclinical testing that more accurately predicts drug efficacy in the clinic. Autochthonous mouse models that have been employed for testing are typically predisposed to cancer because of a genetic alteration in a single gene and all the cells in the tumor harbor the mutation. Treatments designed to target a specific alteration often elicit dramatic results because most, if not all cells, in a tumor respond. Our results indicate that tumor response will likely depend on the clonal architecture of a tumor especially when a tumor is composed of susceptible and resistant cells. We demonstrated that the response to low‐dose aspirin was impacted by the genotype of neoplastic cells of the tumor.

Our study has limitations. The Min mouse is a model of familial adenomatous polyposis which is a hereditary form of colorectal cancer. Data are steadily accruing indicating that tumors with a multi‐ancestral architecture can form in patients with sporadic disease. Our findings are likely not only relevant to hereditary forms of colorectal cancers. Another limitation of our study is that Cre recombination is not equally efficient for all transgenes in all cells.^54^ However, tumors labeled with tdTomato were similar in frequency and size to tumors from control animals that lacked Pik3ca* expression, and tumors labeled with EGFP were significantly larger and more invasive than those that lacked EGFP expression. In addition, the patchwork of red and green cells remains stable across age groups, and cells were never observed to change color after tracking 299 organoids for 7 days in in vitro experiments (this study) or in previous long‐term longitudinal in vivo experiments, indicating that once a clone is labeled with EGFP or tdTomato that label is not likely to change.^32^ Therefore, regardless of the genetic status of the individual clones, heterotypic tumors were likely truly multi‐ancestral in origin.

In the age of genomics and sequencing, a major focus of cancer research is upon identifying the genetic aberrations within clones. This focus emphasizes genetic heterogeneity as a potential mechanism for tumor adaptability and survivability; in the presence of insults such as immune system activation, hypoxic conditions, or chemotherapy, a genetically heterogeneous tumor has numerous opportunities to contain a pre‐existing resistant clone. In this view of tumor heterogeneity, each clone exists independent of all other clones. Another component of tumor heterogeneity, however, is cooperation between clones.^55^ Just as intercellular interactions are critical to angiogenesis and immune escape, interclonal interactions likely contribute to tumor malignancy.^10^ Identifying and understanding the biological mechanism and repercussions of these interclonal interactions might therefore lead to the discovery of effective novel prevention and treatment strategies.

## CONFLICT OF INTEREST

The authors have no conflict of interest to declare.

## AUTHORS' CONTRIBUTIONS

All authors had full access to the data in the study and take responsibility for the integrity of the data and the accuracy of the data. *Conceptualization*, *Data Curation*, *Formal Analysis*, *Investigation*, *Methodology*, *Supervision*, *Writing—Original Draft*, A.A.L.; *Investigation*, *Methodology*, B.J.G.; *Investigation*, *Methodology*, A.M.W.; *Investigation*, *Methodology*, E.R.W; *Investigation*, *Methodology*, S.W.; *Investigation*, *Methodology*, C.D.Z.; *Formal Analysis*, *Investigation*, *Writing—Review & Editing*, K.A.M.; *Conceptualization*, *Formal Analysis*, *Investigation*, *Writing—Review & Editing*, D.A.D.; *Funding Acquisition*, *Investigation*, N.K.; *Investigation*, *Methodology*, Q.R.; *Conceptualization*, *Investigation*, *Methodology*, *Writing—Review & Editing*, C.K.S.; *Investigation*, *Methodology*, A.R.S.; *Formal Analysis*, *Investigation*, *Methodology*, *Writing—Review & Editing*, D.M.A.; *Data Curation*, *Visualization*, *Writing—Review & Editing*, L.C.; *Funding Acquisition*, *Investigation*, *Methodology*, H.M.; *Formal Analysis*, *Supervision*, *Writing—Review & Editing*, M.A.N.; *Conceptualization*, *Formal Analysis*, *Funding Acquisition*, *Investigation*, *Methodology*, *Project Administration*, *Resources*, *Writing—Review & Editing*, R.B.H.

## ETHICAL STATEMENT

Animal studies were conducted under protocols approved by the School of Medicine and Public Health Institutional Animal Care and Use Committee at the University of Wisconsin in compliance with policies established by the Office of Laboratory Animal Welfare at the National Institutes of Health.

## Supporting information


**Appendix S1**: Supporting Information

## Data Availability

The data that support the findings of this study are available from the corresponding author upon reasonable request.

## References

[1] Anaka M , Hudson C , Lo PH , et al. Intratumoral genetic heterogeneity in metastatic melanoma is accompanied by variation in malignant behaviors. BMC Med Genomics. 2013;6:40. 24119551 10.1186/1755-8794-6-40 PMC3852494

[2] Medeiros BC , Othus M , Fang M , Appelbaum FR , Erba HP . Cytogenetic heterogeneity negatively impacts outcomes in patients with acute myeloid leukemia. Haematologica. 2015;100(3):331‐335. 25527568 10.3324/haematol.2014.117267 PMC4349271

[3] Mengelbier LH , Karlsson J , Lindgren D , et al. Intratumoral genome diversity parallels progression and predicts outcome in pediatric cancer. Nat Commun. 2015;6:6125. 25625758 10.1038/ncomms7125

[4] Mroz EA , Tward AD , Hammon RJ , Ren Y , Rocco JW . Intra‐tumor genetic heterogeneity and mortality in head and neck cancer: analysis of data from the cancer genome atlas. PLoS Med. 2015;12(2):e1001786. 25668320 10.1371/journal.pmed.1001786 PMC4323109

[5] Landau DA , Carter SL , Stojanov P , et al. Evolution and impact of subclonal mutations in chronic lymphocytic leukemia. Cell. 2013;152(4):714‐726. 23415222 10.1016/j.cell.2013.01.019 PMC3575604

[6] Ding L , Ley TJ , Larson DE , et al. Clonal evolution in relapsed acute myeloid leukaemia revealed by whole‐genome sequencing. Nature. 2012;481(7382):506‐510. 22237025 10.1038/nature10738 PMC3267864

[7] Anderson K , Lutz C , van Delft FW , et al. Genetic variegation of clonal architecture and propagating cells in leukaemia. Nature. 2011;469(7330):356‐361. 21160474 10.1038/nature09650

[8] Cleary AS , Leonard TL , Gestl SA , Gunther EJ . Tumour cell heterogeneity maintained by cooperating subclones in Wnt‐driven mammary cancers. Nature. 2014;508(7494):113‐117. 24695311 10.1038/nature13187 PMC4050741

[9] Wu M , Pastor‐Pareja JC , Xu T . Interaction between Ras(V12) and scribbled clones induces tumour growth and invasion. Nature. 2010;463(7280):545‐548. 20072127 10.1038/nature08702 PMC2835536

[10] Marusyk A , Tabassum DP , Altrock PM , Almendro V , Michor F , Polyak K . Non‐cell‐autonomous driving of tumour growth supports sub‐clonal heterogeneity. Nature. 2014;514(7520):54‐58. 25079331 10.1038/nature13556 PMC4184961

[11] Kreso A , O'Brien CA , van Galen P , et al. Variable clonal repopulation dynamics influence chemotherapy response in colorectal cancer. Science. 2013;339(6119):543‐548. 23239622 10.1126/science.1227670 PMC9747244

[12] Lengauer C , Kinzler KW , Vogelstein B . Genetic instabilities in human cancers. Nature. 1998;396(6712):643‐649. 9872311 10.1038/25292

[13] Boland CR , Thibodeau SN , Hamilton SR , et al. A National Cancer Institute workshop on microsatellite instability for cancer detection and familial predisposition: development of international criteria for the determination of microsatellite instability in colorectal cancer. Cancer Res. 1998;58(22):5248‐5257. 9823339

[14] Muto T , Bussey HJ , Morson BC . The evolution of cancer of the colon and rectum. Cancer. 1975;36(6):2251‐2270. 1203876 10.1002/cncr.2820360944

[15] Vogelstein B , Fearon ER , Hamilton SR , et al. Genetic alterations during colorectal‐tumor development. N Engl J Med. 1988;319(9):525‐532. 2841597 10.1056/NEJM198809013190901

[16] Baca SC , Prandi D , Lawrence MS , et al. Punctuated evolution of prostate cancer genomes. Cell. 2013;153(3):666‐677. 23622249 10.1016/j.cell.2013.03.021 PMC3690918

[17] Cross W , Graham TA , Wright NA . New paradigms in clonal evolution: punctuated equilibrium in cancer. J Pathol. 2016;240(2):126‐136. 27282810 10.1002/path.4757

[18] Sottoriva A , Kang H , Ma Z , et al. A Big Bang model of human colorectal tumor growth. Nat Genet. 2015;47(3):209‐216. 25665006 10.1038/ng.3214 PMC4575589

[19] Parsons BL . Multiclonal tumor origin: evidence and implications. Mutat Res. 2018;777:1‐18. 10.1016/j.mrrev.2018.05.001 30115427

[20] Beutler E . Multicentric origin of colon carcinoma. Science. 1984;224(4649):630‐631. 6710162 10.1126/science.6710162

[21] Hsu SH , Luk GD , Krush AJ , Hamilton SR , Hoover HH Jr . Multiclonal origin of polyps in Gardner syndrome. Science. 1983;221(4614):951‐953. 6879192 10.1126/science.6879192

[22] Novelli MR , Williamson JA , Tomlinson IP , et al. Polyclonal origin of colonic adenomas in an XO/XY patient with FAP. Science. 1996;272(5265):1187‐1190. 8638166 10.1126/science.272.5265.1187

[23] Thirlwell C , Will OC , Domingo E , et al. Clonality assessment and clonal ordering of individual neoplastic crypts shows polyclonality of colorectal adenomas. Gastroenterology. 2010;138(4):1441‐1454. 20102718 10.1053/j.gastro.2010.01.033

[24] Merritt AJ , Gould KA , Dove WF . Polyclonal structure of intestinal adenomas in ApcMin/+ mice with concomitant loss of Apc+ from all tumor lineages. Proc Natl Acad Sci USA. 1997;94(25):13927‐13931. 9391129 10.1073/pnas.94.25.13927 PMC28409

[25] Thliveris AT , Clipson L , White A , et al. Clonal structure of carcinogen‐induced intestinal tumors in mice. Cancer Prev Res. 2011;4(6):916‐923. 10.1158/1940-6207.CAPR-11-0022 PMC3220275 21636550

[26] Thliveris AT , Halberg RB , Clipson L , et al. Polyclonality of familial murine adenomas: analyses of mouse chimeras with low tumor multiplicity suggest short‐range interactions. Proc Natl Acad Sci USA. 2005;102(19):6960‐6965. 15870186 10.1073/pnas.0502662102 PMC1100801

[27] Thliveris AT , Schwefel B , Clipson L , et al. Transformation of epithelial cells through recruitment leads to polyclonal intestinal tumors. Proc Natl Acad Sci USA. 2013;110(28):11523‐11528. 23798428 10.1073/pnas.1303064110 PMC3710880

[28] Novelli MR , Wasan H , Rosewell I , et al. Tumor burden and clonality in multiple intestinal neoplasia mouse/normal mouse aggregation chimeras. Proc Natl Acad Sci USA. 1999;96(22):12553‐12558. 10535960 10.1073/pnas.96.22.12553 PMC22985

[29] Powell AE , Vlacich G , Zhao ZY , et al. Inducible loss of one Apc allele in Lrig1‐expressing progenitor cells results in multiple distal colonic tumors with features of familial adenomatous polyposis. Am J Physiol Gastrointest Liver Physiol. 2014;307(1):G16‐G23. 24833705 10.1152/ajpgi.00358.2013 PMC4080164

[30] Fischer JM , Schepers AG , Clevers H , Shibata D , Liskay RM . Occult progression by Apc‐deficient intestinal crypts as a target for chemoprevention. Carcinogenesis. 2014;35(1):237‐246. 23996931 10.1093/carcin/bgt296 PMC3871938

[31] Schepers AG , Snippert HJ , Stange DE , et al. Lineage tracing reveals Lgr5+ stem cell activity in mouse intestinal adenomas. Science. 2012;337(6095):730‐735. 22855427 10.1126/science.1224676

[32] Zahm CD , Szulczewski JM , Leystra AA , et al. Advanced intestinal cancers often maintain a multi‐ancestral architecture. PLoS One. 2016;11(2):e0150170. 26919712 10.1371/journal.pone.0150170 PMC4769224

[33] Parsons BL . Many different tumor types have polyclonal tumor origin: evidence and implications. Mutat Res. 2008;659(3):232‐247. 18614394 10.1016/j.mrrev.2008.05.004

[34] Yu C , Yu J , Yao X , et al. Discovery of biclonal origin and a novel oncogene SLC12A5 in colon cancer by single‐cell sequencing. Cell Res. 2014;24(6):701‐712. 24699064 10.1038/cr.2014.43 PMC4042168

[35] Kosmidou V , Oikonomou E , Vlassi M , et al. Tumor heterogeneity revealed by KRAS, BRAF, and PIK3CA pyrosequencing: KRAS and PIK3CA intratumor mutation profile differences and their therapeutic implications. Hum Mutat. 2014;35(3):329‐340. 24352906 10.1002/humu.22496

[36] Samuels Y , Wang Z , Bardelli A , et al. High frequency of mutations of the PIK3CA gene in human cancers. Science. 2004;304(5670):554. 15016963 10.1126/science.1096502

[37] Jones S , Chen WD , Parmigiani G , et al. Comparative lesion sequencing provides insights into tumor evolution. Proc Natl Acad Sci USA. 2008;105(11):4283‐4288. 18337506 10.1073/pnas.0712345105 PMC2393770

[38] Leystra AA , Deming DA , Zahm CD , et al. Mice expressing activated PI3K rapidly develop advanced colon cancer. Cancer Res. 2012;72(12):2931‐2936. 22525701 10.1158/0008-5472.CAN-11-4097 PMC3645915

[39] Deming DA , Leystra AA , Nettekoven L , et al. PIK3CA and APC mutations are synergistic in the development of intestinal cancers. Oncogene. 2014;33(17):2245‐2254. 23708654 10.1038/onc.2013.167 PMC3883937

[40] Yueh AE , Payne SN , Leystra AA , et al. Colon cancer tumorigenesis initiated by the H1047R mutant PI3K. PLoS One. 2016;11(2):e0148730. 26863299 10.1371/journal.pone.0148730 PMC4749659

[41] Vivanco I , Sawyers CL . The phosphatidylinositol 3‐kinase AKT pathway in human cancer. Nat Rev Cancer. 2002;2(7):489‐501. 12094235 10.1038/nrc839

[42] Wong MH , Saam JR , Stappenbeck TS , Rexer CH , Gordon JI . Genetic mosaic analysis based on Cre recombinase and navigated laser capture microdissection. Proc Natl Acad Sci USA. 2000;97(23):12601‐12606. 11050178 10.1073/pnas.230237997 PMC18810

[43] Muzumdar MD , Tasic B , Miyamichi K , Li L , Luo L . A global double‐fluorescent Cre reporter mouse. Genesis. 2007;45(9):593‐605. 17868096 10.1002/dvg.20335

[44] Moser AR , Pitot HC , Dove WF . A dominant mutation that predisposes to multiple intestinal neoplasia in the mouse. Science. 1990;247(4940):322‐324. 2296722 10.1126/science.2296722

[45] Srinivasan L , Sasaki Y , Calado DP , et al. PI3 kinase signals BCR‐dependent mature B cell survival. Cell. 2009;139(3):573‐586. 19879843 10.1016/j.cell.2009.08.041 PMC2787092

[46] Miskovitz P , ed. Colonoscopy. London, UK: InTech; 2011.

[47] Xue X , Shah YM . In vitro organoid culture of primary mouse colon tumors. J Vis Exp. 2013;75:e50210. 10.3791/50210 PMC3684363 23711911

[48] Chen Z , Wang C , Dong H , et al. Aspirin has a better effect on PIK3CA mutant colorectal cancer cells by PI3K/Akt/raptor pathway. Mol Med. 2020;26(1):14. 32000660 10.1186/s10020-020-0139-5 PMC6993447

[49] Liao X , Lochhead P , Nishihara R , et al. Aspirin use, tumor PIK3CA mutation, and colorectal‐cancer survival. N Engl J Med. 2012;367(17):1596‐1606. 23094721 10.1056/NEJMoa1207756 PMC3532946

[50] Newton MA , Clipson L , Thliveris AT , Halberg RB . A statistical test of the hypothesis that polyclonal intestinal tumors arise by random collision of initiated clones. Biometrics. 2006;62(3):721‐727. 16984313 10.1111/j.1541-0420.2006.00522.x

[51] Beutler E , Collins Z , Irwin LE . Value of genetic variants of glucose‐6‐phosphate dehydrogenase in tracing the origin of malignant tumors. N Engl J Med. 1967;276(7):389‐391. 6017245 10.1056/NEJM196702162760706

[52] Hadac JN , Leystra AA , Paul Olson TJ , et al. Colon tumors with the simultaneous induction of driver mutations in APC, KRAS, and PIK3CA still Progress through the adenoma‐to‐carcinoma sequence. Cancer Prev Res. 2015;8(10):952‐961. 10.1158/1940-6207.CAPR-15-0003 PMC4596777 26276752

[53] Waclaw B , Bozic I , Pittman ME , Hruban RH , Vogelstein B , Nowak MA . A spatial model predicts that dispersal and cell turnover limit intratumour heterogeneity. Nature. 2015;525(7568):261‐264. 26308893 10.1038/nature14971 PMC4782800

[54] Vooijs M , Jonkers J , Berns A . A highly efficient ligand‐regulated Cre recombinase mouse line shows that LoxP recombination is position dependent. EMBO Rep. 2001;2(4):292‐297. 11306549 10.1093/embo-reports/kve064 PMC1083861

[55] Tabassum DP , Polyak K . Tumorigenesis: it takes a village. Nat Rev Cancer. 2015;15(8):473‐483. 26156638 10.1038/nrc3971

