# Supplementary material for: Multi‐ancestral origin of intestinal tumors: Impact on growth, progression, and drug efficacy
Source: Cancer Rep (Hoboken). 2021 Jul 10;5(2):e1459. doi: 10.1002/cnr2.1459 (PMC8842699; doi:10.1002/cnr2.1459)
Supplement: Supplementary file 1 — Appendix S1: Supporting Information [file CNR2-5-e1459-s001.pdf]

$$\mathit{Perimeter(green)}$$

Green patch perimeter is directly proportional to the number of green crypts adjacent to crypts of another color.

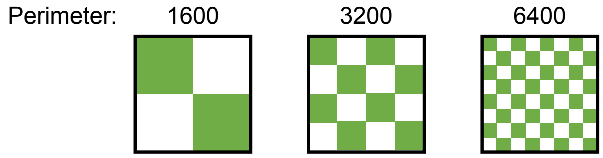

$$\frac{\mathit{Perimeter(green)}}{\sqrt{\mathit{Area(tissue)}}}$$

Perimeter must be normalized to tissue area to avoid giving falsely higher variegation scores to higher resolution images or larger tissues.

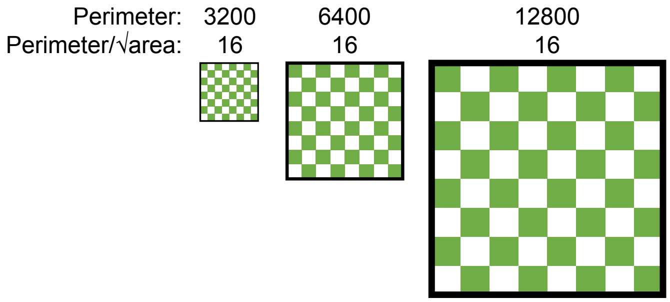

Variegation Score =

$$\frac{\mathit{Perimeter(green)} - (\mathit{Proportion(green)} \times \mathit{Perimeter(tissue)})}{\sqrt{\mathit{Area(tissue)}}}$$

Green crypts on the tissue perimeter do not have an adjacent crypt of another color; their outer edges must be subtracted to avoid giving falsely higher scores to tissues with more green.

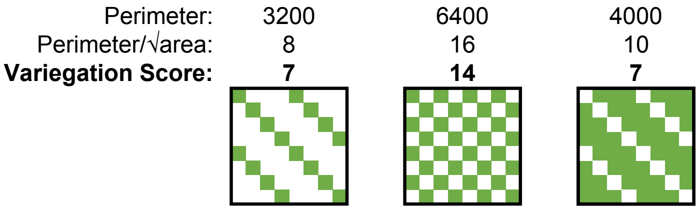

Figure S1. Development of the variegation score. The equation (left) was developed to represent the number of crypts that are adjacent to a crypt of a different color. In this way, a high variegation score would represent a high power to detect multi-ancestral tumors. Images with varying patchwork (right) were used to confirm the equation works as expected.

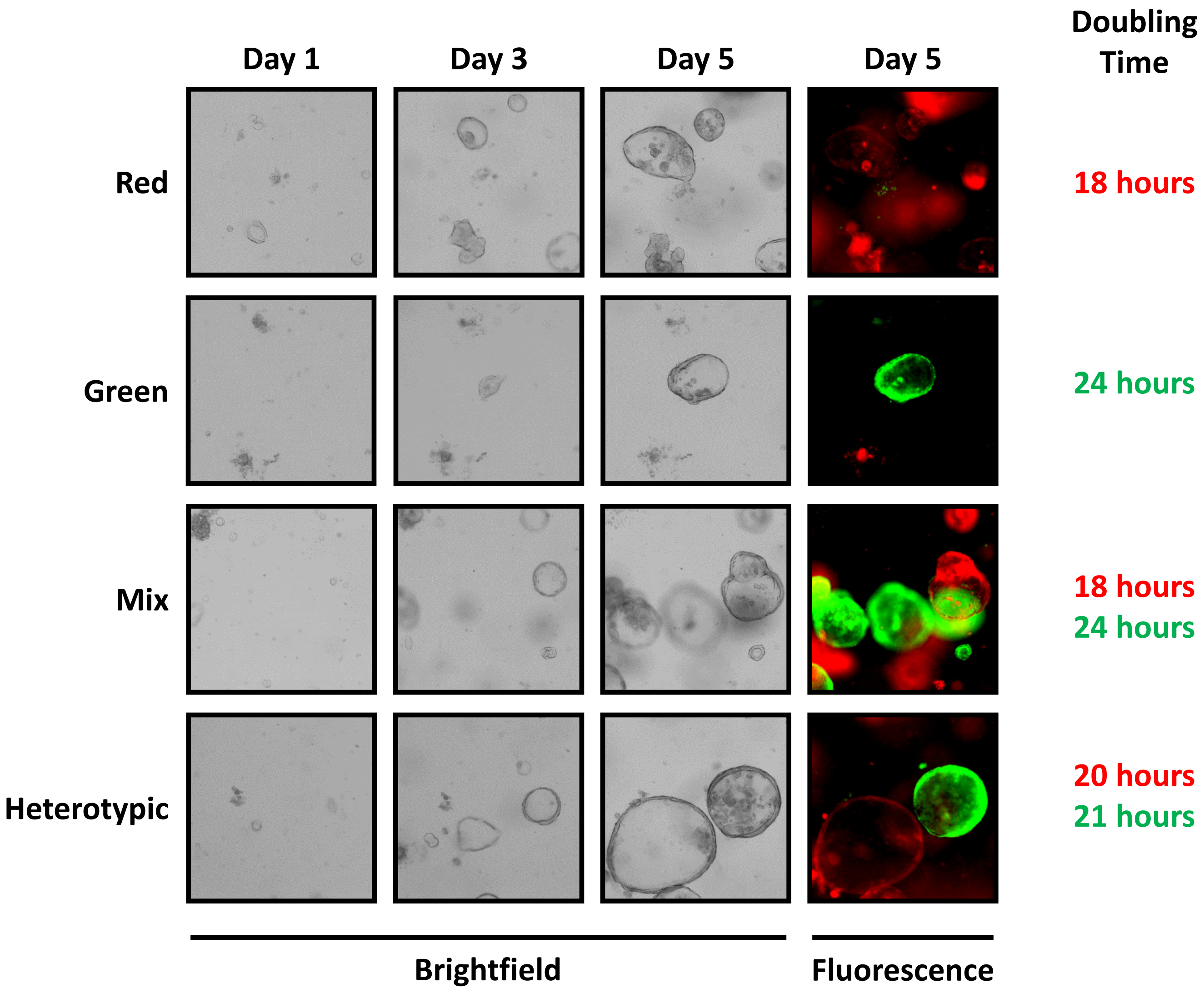

Figure S2. Organoids can be generated from homotypic red, homotypic green, and heterotypic tumors from experimental mice. Intestinal tumors were removed from experimental mice and minced with a razor blade, digested to generate a single cell suspension, and cultured to grow organoids. Cells from homotypic red tumors and cells from homotypic green tumors formed wholly red or wholly green organoids, respectively. The maximum diameter for each of the 299 organoids was determined each day. The time that was required for the average maximum diameter to double was faster for those derived from homotypic red tumors as compared to those derived from homotypic green tumors (18 hours versus 24 hours). These specific maximum diameter doubling times did not change when the cells from homotypic red tumors were mixed at a 1:1 ratio with cells from homotypic green tumors. Cells from heterotypic tumors also formed wholly red and wholly green organoids. Interestingly, the maximum diameter doubling time of red organoids from heterotypic tumors was 20 hours and that for green organoids was 21 hours.

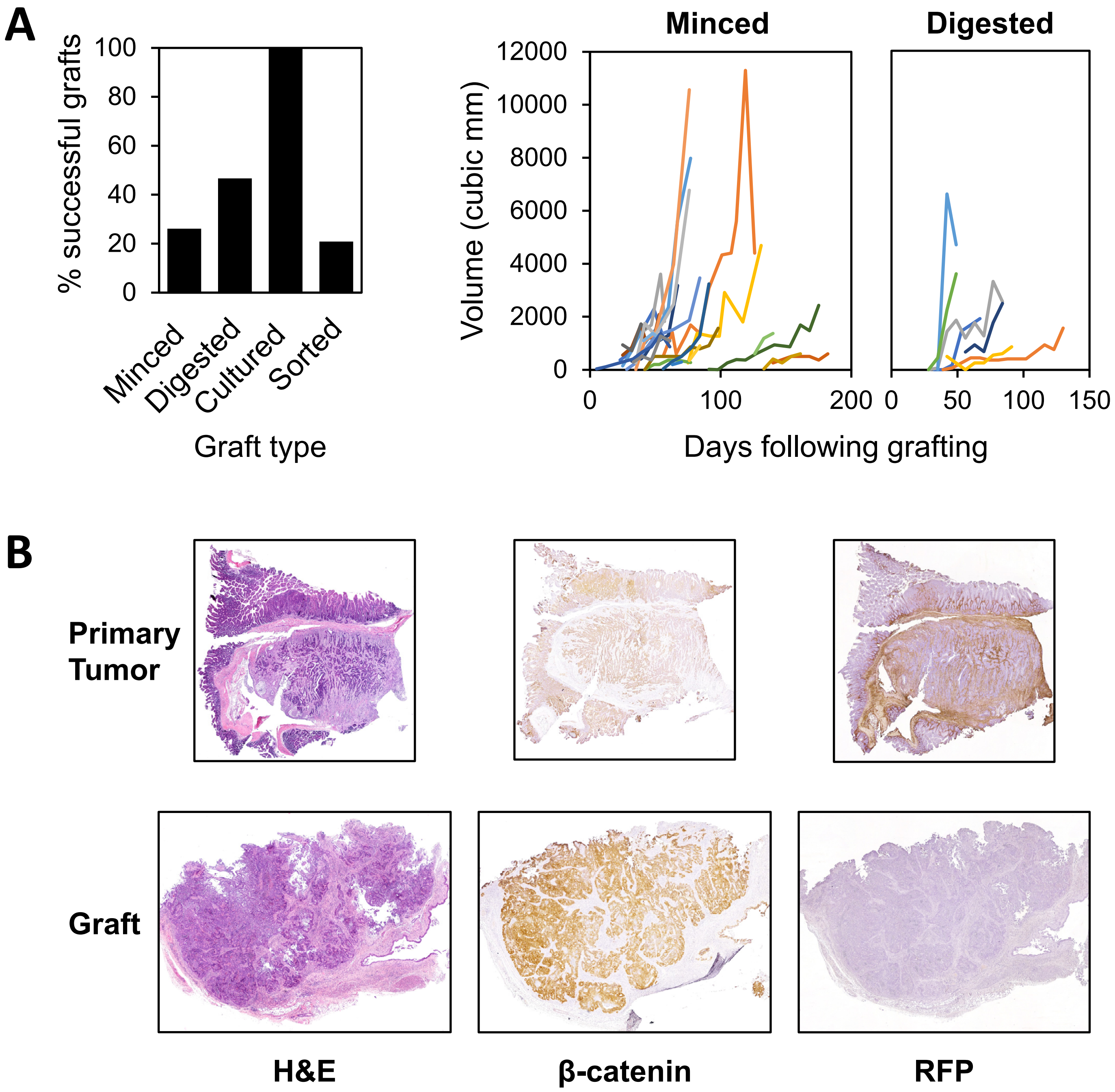

Figure S3. Homotypic red, homotypic green, and heterotypic tumors from experimental mice can be successfully grafted onto the flank of recipient mice. Intestinal tumors were removed and minced with a razor blade, digested to generate single cell suspension, or cultured to grow organoids prior to injection into the flank of a recipient mouse. In some cases, successful grafts were removed, digested, and sorted to isolate either red or green cells. The sorted cells were grafted into a new recipient. Regardless the method used to prepare samples, tumors formed from grafts albeit the success rated varied from 21% with sorted cells to 100% with cultured organoids (A; left panel). In total, grafts were confirmed by histology or cell sorting to be derived from two homotypic red tumors, seven homotypic green tumors, and five heterotypic tumors. The growth of the tumor grafts was highly variable (A; right panel). This variability is comparable to growth of colon tumors in mice or humans. An example of a primary tumor and the resulting successful graft is shown (B). The tumor was highly invasive and composed entirely of green neoplastic cells with many exhibiting nuclear β-catenin, a tumor marker in the intestine. Graft tumors were quite similar to the primary tumors from which they originated, with the exception that the stromal cells within the graft tumors came from the recipient mice, given that these cells lacked RFP. Thus, growth rates of colon tumor cells might be related to both the mutations present and the clonal architecture of the tumor from which they are derived.

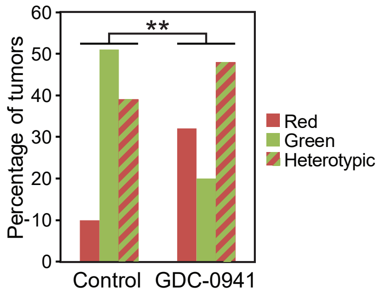

Figure S4. Multi-ancestral tumors might be more resistant to PI3K inhibition than homotypic counterparts. After treatment, mice were euthanized to score the number of homotypic red, homotypic green, and heterotypic tumors along the entire length of intestinal tract.

Supplementary Methods

Breeding Scheme

FVB/N- and C57BL/6J-*Tg*$^{(Fabp1\text{-}Cre)1}$*Jig* mice homozygous for the Fabp1-Cre transgene were crossed with B6.129(Cg)-*Gt(ROSA)26Sor*$^{tm4(ACTB\text{-}tdTomato,\text{-}EGFP)Luo}$*/J* mice homozygous for the mT/mG transgene to generate (FVBxB6)F1 Fabp1-Cre mT/mG mice and a B6.Fabp1-Cre mT/mG line, respectively. The B6.Fabp1-Cre mT/mG line was maintained by sibling crosses for no more than ten generations prior to backcrossing to C57BL/6J mice obtained from The Jackson Laboratory.

C57BL/6-*Gt(ROSA)26Sor*$^{tm7(Pik3ca*,EGFP)Rsky}$/J mice, which were homozygous for the Pik3ca* transgene, were crossed with B6.*Apc*^{Min} mice, which were heterozygous for the *Min* allele of the *Adenomatous polyposis coli* gene, to generate a B6.Pik3ca**Apc*^{Min} line. These mice were maintained by sibling crosses for no more than ten generations prior to backcrossing to C57BL/6J mice obtained from The Jackson Laboratory or newly imported C57BL/6-*Gt(ROSA)26Sor*$^{tm7(Pik3ca*,EGFP)Rsky}$/J mice.

(FVBxB6)F1 Fabp1-Cre mT/mG and B6.Fabp1-Cre mT/mG were crossed with B6.Pik3ca**Apc*^{Min} to generate (FVBxB6)xB6 N2 and B6.Fabp1-Cre mT/mG Pik3ca**Apc*^{Min} mice respectively. Resulting progeny that were hemizygous for all transgenes and heterozygous for the *Apc*^{Min} allele were experimental animals while littermate controls lacked one or more of the transgenes or were wild type at the *Apc* locus.

Image Processing Details

Briefly, the macro searched a directory for Zeiss image files (ZVI) and converted them to TIFF files using the LOCI Bio-Formats Importer. It then identified all TIFF files, set an autothreshold, converted to binary, and measured the area and perimeter of patches of pixels that represented green fluorescence and the area and perimeter of patches of pixels that represented red fluorescence. Areas of the image containing no tissue were filled with gray. The result was a binary image in which white represented tissue expressing tdTomato and black represented tissue expressing EGFP.

Table S1. Tumor number data shown in Figure 3A.

PI3K activated	N of mice	N of tumors, mean ± SEM					
		SI-1	SI-2	SI-3	SI-4	CO	Total
No	10	1.3 ± 0.6	1.8 ± 1.4	1.5 ± 1.0	2.4 ± 1.8	0.2 ± 0.1	7.2 ± 4.7
Yes	12	1.6 ± 0.7	2.3 ± 0.4	2.9 ± 0.7	7.8 ± 1.2	2.7 ± 0.7	17.3 ± 2.1

Differences in tumor numbers for total, S4 and CO are statistically significant ($p = 0.001$, $p = 0.004$ and 0.004, respectively, two-sided Wilcoxon rank sum test).

Table S2. Tumor numbers by tumor color for 8 mice with PI3K activated and tumor color data available. Data for heterotypic, homotypic green and homotypic red tumors shown in Figure 3A.

Tumor color	N of tumors, mean ± SEM					
	SI-1	SI-2	SI-3	SI-4	CO	Total
Heterotypic	0	0	0	1.8 ± 0.5	1.0 ± 0.6	2.8 ± 0.8
Homotypic green	0	0	0.3 ± 0.3	1.6 ± 0.8	1.3 ± 0.6	3.1 ± 1.4
Homotypic red	1.4 ± 0.8	2.1 ± 0.5	1.4 ± 1.1	1.0 ± 0.3	0	5.9 ± 1.3
ND	0	0	0.3 ± 0.3	0.8 ± 0.5	0.6 ± 0.4	1.6 ± 0.7
Total	1.4 ± 0.8	2.1 ± 0.5	1.9 ± 1.1	5.1 ± 1.5	2.9 ± 1.0	13.4 ± 3.0

No statistically significant differences were found between homotypic red tumors (this table) and PI3K non-activated tumors (Table S1) for any of the intestinal sections or the total multiplicity (p-values > 0.05, two-sided Wilcoxon rank sum tests). ND, not determined.

Table S3. Tumor size data shown in Figure 3B.

PI3K activated	N of mice	Tumor size (mm), mean ± SEM					
		SI-1	SI-2	SI-3	SI-4	CO	Total
No	8	1.4 ± 0.2	1.0 ± 0.1	0.8 ± 0.1	0.9 ± 0.1	2.9 ± 2.0	1.0 ± 0.1
Yes	12	1.2 ± 0.1	1.1 ± 0.1	1.3 ± 0.3	2.9 ± 0.2	3.1 ± 0.4	2.3 ± 0.1

Differences in tumor size for S4 are statistically significant ($p < 0.001$, Wilcoxon rank sum test).

Table S4. Tumor size for 8 mice with PI3K activated and tumor color data available. Data shown in Figure 3B.

Tumor color	Tumor size (mm), mean ± SEM					
	SI-1	SI-2	SI-3	SI-4	CO	Total
Heterotypic	NA	NA	NA	3.9 ± 0.7	3.3 ± 0.5	3.6 ± 0.5
Homotypic green	NA	NA	0.5 ± 0.0	2.5 ± 0.5	1.8 ± 0.5	2.1 ± 0.3
Homotypic red	1.1 ± 0.1	1.1 ± 0.1	0.7 ± 0.1	1.4 ± 0.3	NA	1.1 ± 0.1

Heterotypic tumors were significantly larger compared to homotypic green in CO ($p = 0.03$) and compared to PI3K non-activated (Table S3, $p < 0.001$) and homotypic red in S4 ($p = 0.005$), but not compared to homotypic green in S4 ($p = 0.16$). No statistically significant differences were found between homotypic red tumors (this table) and PI3K non-activated tumors (Table S3) for any of the intestinal sections or average size for the total (p-values > 0.05, Wilcoxon rank sum test). Differences in size between homotypic green tumors and PI3K non-activated tumors (Table S3) were statistically significant for S4 and total ($p < 0.01$ for each). Two-sided Wilcoxon rank sum tests were used. NA, not applicable.

Table S5. Tumor numbers from Pik3ca*+ $Apc^{Min/+}$ mice.

		N of tumors, mean ± SEM					
mT/mG activation	N of mice	Total	SI-1	SI-2	SI-3	SI-4	CO
Yes	9	14.7 ± 2.9	1.4 ± 0.7	2.0 ± 0.5	5.9 ± 1.5	5.8 ± 4.4	2.9 ± 0.9
No	3	25.0 ± 11.7	2.0 ± 0.6	3.0 ± 3.0	4.3 ± 2.3	13.7 ± 5.2	2.0 ± 1.2

For comparisons of tumor numbers by section (or “Total”) for mice with TG expression versus those without mT/mG activation, $p \geq 0.05$ using two-sided Wilcoxon rank sum tests.
